# Antiresorptive Agents Increase the Effects of Exercise on Preventing Postmenopausal Bone Loss in Women: A Meta-Analysis

**DOI:** 10.1371/journal.pone.0116729

**Published:** 2015-01-23

**Authors:** Renqing Zhao, Zhengang Xu, Meihua Zhao

**Affiliations:** College of Physical Education and Health Sciences, Zhejiang Normal University, Jinhua, Zhejiang, China; Oklahoma State University, UNITED STATES

## Abstract

**Background and Objectives:**

It remains unknown whether the combination of antiresorptive agents and exercise would generate additive effects on bone mineral density (BMD) in postmenopausal women, though their separate roles in preventing bone loss have been well established. This meta-analysis aimed to evaluate the combined impact of antiresorptive treatment and exercise on the lumbar spine and femoral neck BMD in postmenopausal women compared with an exercise-only intervention.

**Methods:**

A systematic literature search of PubMed, EMBASE, SportDiscus and ProQuest up to Jun 2014 was conducted to identify the influence of antiresorptive agents and exercise on BMD in postmenopausal women. The study quality of the included trials was evaluated. The effect sizes were estimated by calculating the standardized mean difference (SMD). Subgroup analyses were conducted by pharmacological regimens and exercise categories.

**Results:**

Nine studies with a total of 1,248 postmenopausal women met the inclusion criteria. The heterogeneity between the studies was evident at the spine (I^2^ = 78.7%) and hip (I^2^ = 41.7%) measurements; random-effects models were used in the data analysis. The pooled effect sizes associated with the combined interventions of antiresorptive agents and exercise were significant at the lumbar spine BMD (SMD = 0.511, 95% CI = 0.118-0.904, *p* = 0.011). Combining hormone replacement therapy (HRT) and exercise training generated greater beneficial effects on lumbar spine (SMD = 0.729, 95% CI = 0.186-1.273, *p* = 0.009) and femoral neck BMD (SMD = 0.220, 95% CI = 0.0110-429, *p* = 0.039) than the exercise-only intervention. Impact exercise was sensitive to antiresorptive agents in preventing postmenopausal bone loss both at the spine (SMD = 1.252, 95%CI = 0.465-2.039, *p* = 0.002) and hips (SMD = 0.414, 95%CI = 0.106-0.723, *p* = 0.008).

**Conclusions:**

Our findings indicate that antiresorptive agents significantly increase the impact of exercise on the prevention of bone loss in postmenopausal women, which implies that the combination of antiresorptive agents and exercise may generate additive effects.

## Introduction

Fracture is a leading cause of morbidity and mortality in postmenopausal women [1]. Exercise [2–5] and antiresorptive agents [6–9], such as estrogen, phytoestrogens and bisphosphonates, are recognized as effective strategies for preventing postmenopausal bone loss and reducing the risks for fracture.

Compelling evidence has demonstrated that exercise training has beneficial effects on skeletal loading sites [3, 10–16]. However, exercise-associated positive effects may be impaired by estrogen deficiency in postmenopausal women because a low estrogen status can decrease the number and function of the estrogen receptor α (ERα) [17–21], through which estrogen regulates the skeletal response to mechanical loading. Currently, estrogen administration is known to partly reverse the decreased osteogenic response to mechanical loading by up-regulating the ERα numbers and activity level [22]. Traditional hormone replacement therapy (HRT) combined with exercise has been recommended to be an optimum intervention for preventing postmenopausal bone loss in several clinical studies [12–15, 23, 24]. However, a possible link with severe side effects prevents many older women from starting HRT and causes them to turn to alternative therapies, such as phytoestrogens. Phytoestrogens are found in plant products, such as soybeans, which are a rich source of isoflavones, genistein and daidzein. Isoflavones are structurally similar to estradiol and act as estrogen antagonists in some cases and estrogen agonists in others by competing with estradiol for estrogen receptor sites [25]. Several studies have reported that isoflavone supplementation and exercise cooperatively inhibited bone loss in female osteoporotic animal models [26, 27] and in postmenopausal women [28]. However, negative findings were recently reported in a randomized clinical trial [29].

Given that bisphosphonates depress bone resorption and exercise increases bone formation, the combination of bisphosphonates and exercise is expected to produce additive effects on BMD [30]. Currently, the combined interventions of antiresorptive agents and exercise have drawn great attention to the maintenance of bone density [12–15, 23, 24, 28–32], but a general consensus is far from determined due to inconsistent results. For the inconsistent findings, wide variation existed in the sample sizes, antiresorptive agent regimens, training frequencies and intensities in the exercise programs and pharmacological interventions. It is necessary to combine the positive and negative outcomes of these studies and employ a meta-analysis to reach some general conclusions about a body of research. This study aimed to examine the additive effects of antiresorptive agents and exercise training for preventing postmenopausal bone loss at the hips and spine.

## Materials and Methods

This meta-analysis was conducted in accordance with PRISMA recommendations and the criteria of the reporting of meta-analysis guidelines [33]. The statistical analysis methods and inclusion criteria were specified and documented in a protocol.

### Search Strategy and Inclusion Criteria

A systematic literature search of PubMed, EMBASE, SportDiscus and ProQuest up to Jan 2014 was conducted to identify all published clinical trials involving the influence of antiresorptive agents and exercise on BMD in postmenopausal women. The terms used for the database searches included “exercise”, “antiresorptive agents”, “hormone replacement therapy”, “estrogen”, “estradiol”, “isoflavone”, “phytoestrogen”, “bisphosphonate”, “alendronate” and “bone mineral density”, and the search was limited to female subjects. We also searched the reference lists of the included papers and conducted a forward search. The inclusion criteria are given in the Table 1. Briefly, the included studies were controlled trials (CTs) or randomized controlled trials (RCTs), which compared the change in BMD between the combined exercise and antiresorptive agent intervention groups and the exercise-only groups in postmenopausal women. The populations of interest were postmenopausal women without regular exercises (less than 2 hours per week) prior to enrollment. The interventions of the included studies were restricted to the combination of exercise and antiresorptive agents, and the duration lasted for at least six months. We included CTs because of the limited number of eligible studies and because long term exercise interventions were frequently available as CTs.

**Table 1:** Inclusion criteria for the trials in the meta-analysis.

Inclusion criterion	Description
Study design	Controlled or randomized controlled trials
Population of interest	Postmenopausal women without disease history or surgical experience affecting bone metabolism.
Exercise experience	Subjects without regular exercise (less than 2 h per week) at least for one year prior to study enrollment.
Interventions	Antiresorptive treatment and exercise training lasting for at least 6 months.
Comparisons	Antiresorptive treatment plus exercise training compared with the exercise-only intervention
Outcome measurements	Absolute or relative changes in BMD at the lumbar spine and femoral neck determined by DXA

BMD: bone mineral density; DXA: dual energy X-ray absorptiometry.

### Data Extraction

All of the data were extracted and reviewed independently by two authors (ZX and MZ). Disagreements were resolved by discussion between the two authors; if no agreement could be reached, a third author would decide. Duplicate published literatures were included only once to ensure that no duplicate data were reviewed in this meta-analysis. The details extracted included: subject characteristics, sample size, exercise interventions (category, intensity, frequency and duration), attrition, compliance, antiresorptive regimens, regions of interest (ROIs) and BMD values with standard deviations (SDs). The primary outcome of the included trials was areal BMD (BMD g/cm^2^), which was assessed by dual energy X-ray absorptiometry (DXA). The absolute and relative changes from baseline to follow-up in BMD, along with the SDs, were used for the meta-analysis. When the changed values were not available from the original publication or the author, these were calculated using baseline and follow-up values. The data extraction followed the methods provided by the Cochrane Reviewers’ Handbook [34].

### Study Quality Assessment

Two authors (ZX and MZ) independently assessed the quality of the included trials using the questionnaire described by Jaded *et al* [35]. The quality scale is a three-item instrument that provides an assessment of bias, specifically focusing on randomization, blinding and withdrawals.

### Statistical Analysis

The primary endpoint of our study was the change in lumbar spine and femoral neck BMD, which was assessed using the standardized mean difference (SMD). The SMD was selected for pooling the intervention effect sizes because of both absolute and relative values used in reporting data in the included studies. We also conducted subgroup analysis by pharmacological regimens and exercise categories to determine whether different pharmacological strategies yielded different impacts on exercise modifying postmenopausal bone loss, and whether different exercise training modes showed different sensitivities to antiresorptive agents in preserving BMD in postmenopausal women.

Our study preferentially used the data analyzed by intention-to-treat (ITT) approach in the original papers to assess the intervention effects; if ITT data were not available, a per-protocol analysis was used in calculating the pooled effect estimates for the combination of the single effects of the trials.

The heterogeneity of results between the studies was determined using Cochran’s Q-test and an alpha value of <0.10 for statistical significance. In addition, I^2^ was used to examine inconsistencies in the study findings. For I^2^, values of <25%, 25% to <50%, 50% to <75% and >75% were considered to be low, moderate, high and very high inconsistency. The tests for the overall effects (Z score) were regarded as significant at p<0.05. STATA version 12 (Stata Corp, TX, USA) was used to perform the meta-analysis and the production of graphics.

## Results

### Study Characteristics

**Figure 1:**
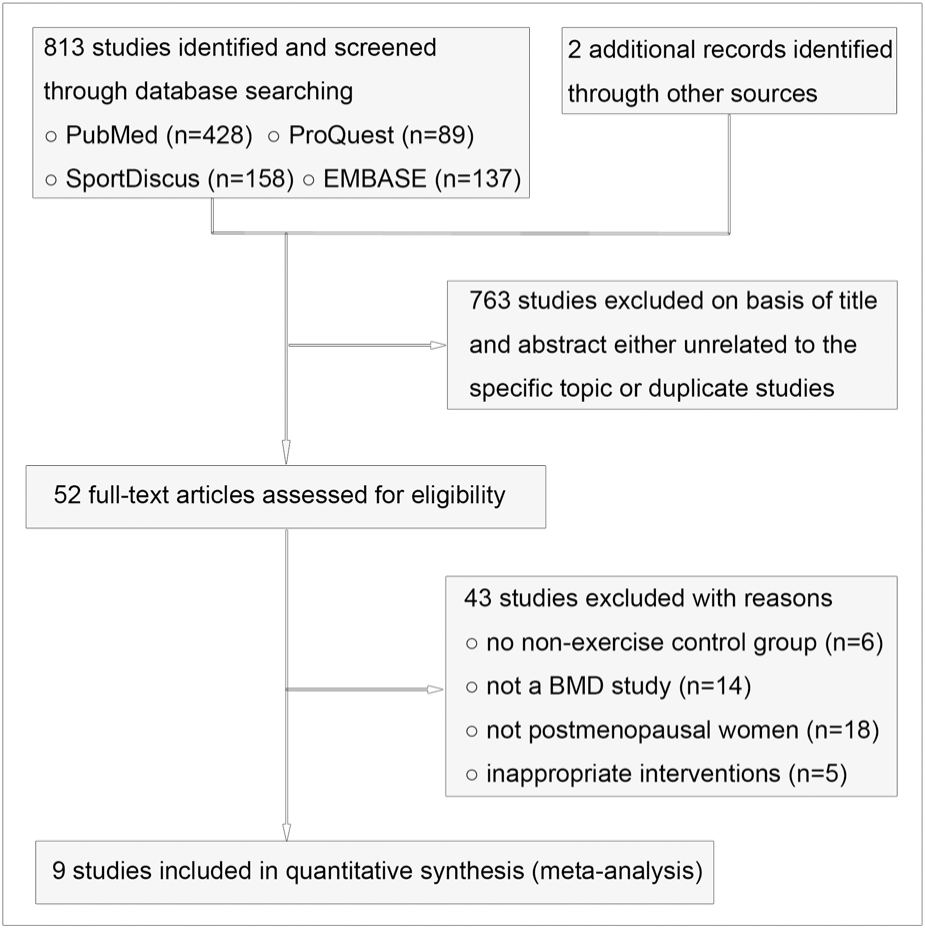
Flow diagram for the selection of the trials.

From the database searches, 815 potential abstracts were identified and screened, of which 763 were excluded because they were unrelated to the specific topic or duplicate trails from different databases (Fig. 1). Fifty-two full-text articles then were reviewed for eligibility. Forty-three of the 52 studies were excluded because of not a BMD study, not postmenopausal women, inappropriate interventions, etc. Nine studies, including 1,248 postmenopausal women (between the ages of 51.8±2.9 and 68.0±3.0 years) in total, met the inclusion criteria (Table 1), amongst which 2 studies were CTs and 7 studies were RCTs (Table 2). Three trials presented ITT data [28–30]; 6 trials only provided per-protocol data. The sample size varied from 32 to 320 participants, and the studies were conducted in Canada [12, 29, 30], Japan [28] and the USA [13–15, 23, 24].

Most of the studies were awarded methodological quality points for randomization and withdrawals. However, only 4 of the 9 studies [12, 28–30] acquired the quality points for blinding. Generally, the quality score of the included trials was relatively low. Four studies [12, 28–30] obtained a quality score of 3 or 5; 3 trials [13, 23, 24] obtained a quality score of 2; 2 trials [14, 15] obtained a quality score of 1 (Table 2).

**Table 2:** Characteristics of the included trials.

Study author and country	Subject age (yrs), [mean ± SD]	Sample size (n)[completed/ dropout]	Pharmacological regimens	Exercise interventions	Device and ROIs	QS
Bassey [12] 1998 Canada	HRT (T = 53.7±3.2, C = 53.4±4.5). nHRT (T = 55.8±3.3, C = 54.9±4.1)	HRT (T = 24/0, C = 22/0). nHRT (T = 45/0, C = 32/0)	Physician prescribed HRT regimens	Five bouts of 10 vertical jumps with arm swing in counter movement, 6 days per week for 12 mos. Compliance: 91%.	DXA: Ls, Fn	3
Chilibeck [30] 2002 Canada	Bisp (T = 55.9±8.3, C = 58.3±6.7). Placebo (T = 56.8±6.3, C = 58.8±5.7)	Bisp (T = 12/3, C = 14/0). Placebo (T = 10/4, C = 12/2)	400 mg/d of etidronate supplement for 14 d, followed by 76 d of 500 mg/d of calcium carbonate	Two sets of 8–10 reps of 5 upper and 4 lower body exercises at 70% 1RM, 3 days per week for 12 mos. Compliance: 77.6%.	DXA: Ls, Fn	3
Chilibeck [29] 2013 Canada	HRT (T = 56.7±6.6, C = 55.8±5.0). Placebo (T = 55.3±6.3, C = 56.4±7.1)	HRT (T = 66/11, C = 66/11). Placebo (T = 71/9, C = 62/3)	165mg isoflavones daily	Two sets of 8 reps of strength training at 80% 1RM, 2 days per week for 24 mos. Compliance: 77%.	DXA: Ls, Fn	5
Going [13] 2003 USA	HRT (T = 54.8±4.0, C = 54.9±5.0). nHRT (T = 55.8±4.7, C = 57.1±5.0)	HRT (T = 71/15, C = 65/8). nHRT (T = 71/20, C = 59/11)	Estrogen, or estrogen and progesterone	Two sets of 6–8 reps of strength training at 70% or 80% 1RM plus high-impact exercises, 3 days per week for 12 mos. Compliance: 79.9%.	DXA: Ls, Fn	2
Kohrt [14] 1995 USA	HRT (T = 66.0±3.0, C = 67.0±3.0). nHRT (T = 65.0±3.0, C = 66.0±3.0)	HRT (T = 8/0, C = 8/0). nHRT (T = 8/0, C = 8/0)	0.625 mg estrogen and 5 mg medroxyprogesterone acetate for 13 consecutive days every third month	Walking, jogging, and/or stair climbing at a heart rate of 126–130 beats/min or 79–80% of maximal heart rate, 3–5days per week for 9 mos. Compliance: 3.3days per week.	DXA: Ls, Fn	1
Kohrt [15] 1998 USA	HRT (T = 66.0±4.0, C = 65.0±3.0). nHRT (T = 66.0±3.0, C = 68.0±3.0)	HRT (T = 16/0, C = 10/0). nHRT (T = 18/0, C = 10/0)	0.625 mg estrogen and 5 mg medroxyprogesterone acetate for 13 consecutive days every third month	Walking, jogging, and/or stair climbing at a heart rate of 126–130 beats/min or 79–80% of maximal heart rate, 3–5days per week for 18 mos. Compliance: 3.3days per week.	DXA: Ls, Fn	1
Maddalozzo [23] 2007 USA	HRT (T = 52.1±3.1, C = 51.8±2.9). nHRT (T = 52.3±3.3, C = 52.5±3.0)	HRT (T = 33/4, C = 34/1). nHRT (T = 29/6, C = 29/5)	0.625 mg conjugated equine estrogen daily	Two sets of 10–12 reps of resistance exercise at 50% 1RM and three sets of 8–12 reps at 60–75% 1 RM, 2 days per week for 12 mos. Compliance: 84.7% and 86.2% for nHRT and HRT plus exercise.	DXA: Ls, Fn	2
Milliken [24] 2003 USA	HRT (54.4 ± 4.4). nHRT (56.9 ± 4.6)	HRT (T = 17/0, C = 21/0). nHRT (T = 25/1, C = 27/3)	Estrogen, or estrogen plus progesterone/ testosterone	Two sets of 6–8 reps of resistance exercises at 70–80% 1RM, plus aerobic weight-bearing exercises, 3 days per week for 12 mos. Compliance: no statement.	DXA: Ls, Fn	2
Wu [28] 2006 Japan	Isoflav (T = 54.4±2.9, C = 53.8±2.9). Placebo (T = 54.9±2.9, C = 55.2±2.8)	Isoflav (T = 30/1, C = 25/8). Placebo (T = 24/7, C = 29/4)	75 mg of isoflavone conjugates/day	One section of 45-min supervised walking exercise at the speed of 5–6 km/h, 3 days per week for 12 mos. Compliance: no statement.	DXA: Ls, Fn	3

T: exercise intervention group; C: control group; RM: repetition maximal; reps: repetitions; yrs: years; mos: months; HRT: hormone replacement therapy; nHRT: non-hormone replacement therapy; SD: standard deviation; Bisp: Bisphosphonate; Isoflav: Isoflavone; Ls: Lumbar spine; Fn: Femoral neck; DXA: dual energy X-ray absorptiometry; ROIs: regions of interest; QS: quality score.

### Antiresorptive Treatment

The antiresorptive regimens included traditional HRTs, isoflavones and bisphosphonates. Six trials [12–15, 23, 24] conducted HRT regimens, but there was no blinding for the estrogen interventions because participants with or without HRT use prior to the start of the study were equally enrolled. Any physician’s prescribed HRT regimens and formulations were accepted (Table 2). Generally, the duration of HRT was relatively short, ranging from 1 to 5.9 years. Two studies [28, 29] conducted isoflavone interventions and used double blinding for the group assignment. The participants in the pharmacological intervention groups were administered isoflavones ranging from 75 mg to 165 mg per day (Table 2). One study [30] used a blinding design for bisphosphonate treatment. The participants were instructed to maintain the same regimen throughout the study and to report any changes if they occurred.

### Exercise Training Interventions

Six hundred and fifty-nine participants, generating 9 study group comparisons, completed a combined intervention of exercise and antiresorptive agents, while 589 control participants, generating a total of 9 study group comparisons, conducted exercise training only. Five trials [13, 23, 24, 29, 36] performed resistance exercise interventions; 4 studies [12, 14, 15, 28] conducted impact exercises including jumping, skipping, jogging, and walking (Table 2). The study durations ranged from 9 to 18 months, with training frequencies of 2–6 times per week. Generally, the participant compliance with the exercise interventions was relatively good, ranging from 65 to 91%. No exercise-related injuries were reported in the included studies.

### Meta-analysis

**Figure 2:**
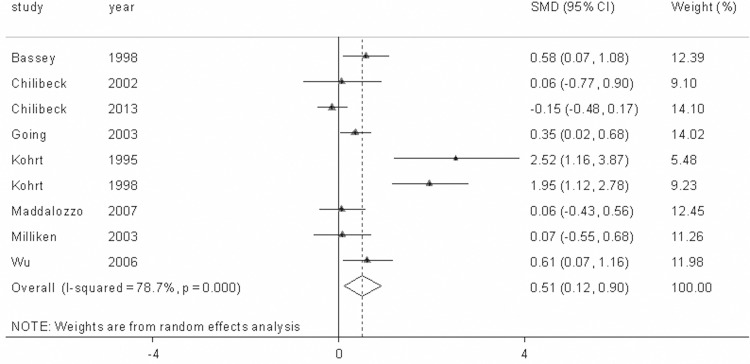
The change in lumbar spine bone mineral density (BMD) with the combined interventions of antiresorptive agents and exercise in all of the included studies. The dotted line represents the mean treatment effect. The diamond denotes the overall treatment effects with 95% confidence intervals (CIs). SMD denotes the standardized mean difference.

**Figure 3:**
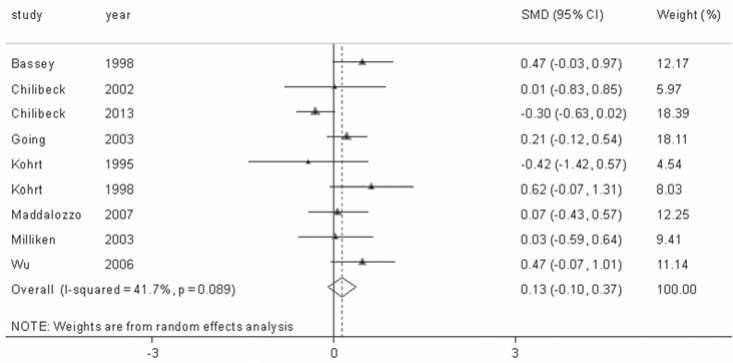
The change in femoral neck bone mineral density (BMD) with the combined interventions of antiresorptive agents and exercise in all of the included studies. The dotted line represents the mean treatment effect. The diamond denotes the overall treatment effects with 95% confidence intervals (CIs). SMD denotes the standardized mean difference.

**The Impact of Combining Antiresorptive Agents and Exercise on BMD**. We conducted 9 study group comparisons to assess the combined influence of antiresorptive agents and exercise training on BMD in postmenopausal women. The included studies showed high levels of heterogeneity at the spine (Q = 37.52, p<0.001, I^2^ = 78.7%) and the hips (Q = 13.72, p = 0.089, I^2^ = 41.7%). Therefore, random-effects models were used in calculating the effect sizes. Our findings indicated that the combination of antiresorptive agents and exercise generated additive effects on lumbar spine BMD (SMD = 0.511, 95% CI = 0.118–0.904, *p* = 0.011) compared with the exercise-only intervention (Fig. 2), whereas the combined intervention of antiresorptive agents and exercise only produced a non-significant positive effect on femoral neck BMD (SMD = 0.135, 95% CI=-0.095–0.365, *p* = 0.251) (Fig. 3).

In the subgroup analysis, we further determined whether different pharmacological strategies yielded different impacts on the exercise modifying postmenopausal bone loss. Six study group comparisons were measured to examine the combined effects of HRT and exercise on BMD in postmenopausal women. The levels of the between-study heterogeneity were high and low at the spine (Q = 26.02, p<0.0001, I^2^ = 80.8%) and hips (Q = 4.60, p = 0.466, I^2^ = 0.0%), respectively. Fixed- and random- effects models were used in the effect size assessment. HRT combined with exercise training generated beneficial effects on lumbar spine (SMD = 0.729, 95% CI = 0.186–1.273, *p* = 0.009) and femoral neck BMD (SMD = 0.220, 95% CI = 0.0110–429, *p* = 0.039) compared with exercise alone (Table 3).

**Table 3:** Primary and subgroup analyses.

Analysis	Studies (*n*)	Participants (*n*)	Heterogeneity (*p* value)	Inconsistency (*I^2^*)	Statistical methods	SMD 95%CI	Test for overall effect
*All Studies*							
Lumbar spine	9	1248	<0.001	78.7%	Random-effects methods	0.511 (0.118 0.904)	z = 2.55 (p = 0.011)
Femoral neck	9	1248	0.089	41.7%	Random-effects methods	0.135 (-0.095 0.365)	z = 1.15 (p = 0.251)
*HRT*							
Lumbar spine	6	1162	<0.001	80.8%	Random-effects methods	0.729 (0.186 1.273)	z = 2.63 (p = 0.009)
Femoral neck	6	1162	0.466	0.0%	Fix-effects methods	0.220 (0.011 0.429)	z = 2.07 (p = 0.039)
*Isoflavone*							
Lumbar spine	2	427	0.018	82.2%	Random-effects methods	0.196 (-0.551 0.942)	z = 0.51 (p = 0.608)
Femoral neck	2	427	0.016	82.8%	Random-effects methods	0.053 (-0.704 0.810)	z = 0.14 (p = 0.891)
*Impact exercise*							
Lumbar spine	4	337	0.002	79.1%	Random-effects methods	1.252 (0.465 2.039)	z = 3.12 (p = 0.002)
Femoral neck	4	337	0.365	5.5%	Fix-effects methods	0.414 (0.106 0.723)	z = 2.63 (p = 0.008)
*Resistance exercise*							
Lumbar spine	5	911	0.333	12.7%	Fix-effects methods	0.083 (-0.110 0.276)	z = 0.84 (p = 0.402)
Femoral neck	5	911	0.284	20.5%	Fix-effects methods	-0.021 (-0.215 0.172)	z = 0.22 (p = 0.828)

There were 2 studies examining the influence of isoflavone and exercise on BMD in postmenopausal women. The levels of heterogeneity between the studies were relatively high, and random-effects models were used in the assessment of effect sizes (Table 3). The combination of isoflavones and exercise training only produced non-significant positive effects on the spine (SMD = 0.196, 95% CI = -0.551–0.942, *p* = 0.608) and hip BMD (SMD = 0.053, 95% CI = -0.704–0.810, *p* = 0.891) in postmenopausal women compared with the exercise-only intervention.

**The Responses of Differing Exercise Training Modes to Antiresorptive Agents in Regulating BMD**. In the subgroup analysis, we also examined whether different exercise training modes (impact exercise *vs* resistance training) showed different sensitivities to antiresorptive agents in preserving BMD in postmenopausal women. Four studies with a population of 337 postmenopausal women and 5 studies including 911 participants conducted impact exercise and resistance training, respectively. In the subgroup analysis of impact exercise, the included studies had high (Q = 14.33, p = 0.002, I^2^ = 79.1%) and low (Q = 3.18, p = 0.365, I^2^ = 5.5%) levels of heterogeneity at the spine and hips, respectively; therefore, fixed- and random- effects models were used in the meta-analysis. The impact exercise intervention was sensitive to antiresorptive treatment in preventing postmenopausal bone loss both at the spine (SMD = 1.252, 95%CI = 0.465–2.039, *p* = 0.002) and hips (SMD = 0.414, 95%CI = 0.106–0.723, *p* = 0.008), whereas the resistance training seemed to be less responsive to the antiresorptive administration in preserving BMD in elderly women (Table 3).

## Discussion

Our meta-analysis aimed to determine whether the combination of antiresorptive agents and exercise training would generate additive effects on preventing postmenopausal bone loss. The systematic searches resulted in 9 clinical trials with a total population of 1,248 postmenopausal women. The findings suggested that combining antiresorptive agents and exercise training generated additive effects on postmenopausal women’s BMD at the spine. Subgroup analyses indicated that HRT significantly increased the impact of exercise on the lumbar spine and femoral neck BMD in postmenopausal women, whereas the combined intervention of isoflavones and exercise did not produce greater effects than exercise alone. Additionally, impact exercise was sensitive to antiresorptive agents in preventing postmenopausal bone loss at the spine and hips.

Considering that the etiology of postmenopausal osteoporosis is complex and multifactorial, combined treatment may generate greater effects. Our findings suggested that antiresorptive agents, including estrogen, isoflavones, and bisphosphonates, significantly increased the effects of exercise on spine BMD in postmenopausal women. The results supported the notion that the combination of antiresorptive agents and exercise generated additive effects on bone loss. The subgroup analyses indicated that HRT significantly increased the effects of exercise training on postmenopausal bone loss at the spine and hips, whereas isoflavones in combination with exercise only generated non-significant effects on bone. However, we should view the non-significant results with caution because of the limited number of studies available for the subgroup analysis of isoflavone interventions. Considering the obvious benefits for preventing bone loss and less adverse effects, isoflavone has been regarded as a promising antiresorptive agent in the prevention of osteoporosis [37, 38]. The combination of isoflavones with exercise is expected to produce additive effects on bone loss. Studies have demonstrated that, in the prevention of bone loss in estrogen-deficient animals, a combined intervention of moderate-intensity exercise and isoflavone administration was more advantageous than either treatment alone [26, 27, 39]. However, in clinical studies, the results were inconsistent. Wu et al [40] found a positive effect of the combined intervention of isoflavones and exercise on bone loss, whereas Chilibeck et al [29] reported negative effects. The different findings probably resulted from the variation that exists in the sample sizes, isoflavone regimens, and training frequencies, duration and intensities. Additionally, only 2 studies included in the assessment of effect sizes may not sufficient to draw a definitive conclusion.

For the reason that HRT probably results in greater risks than benefits [41], many women have been hesitant to start estrogen therapy. Our findings that classic HRT in conjunction with exercise generates greater effects on BMD appear to be limited in preventing bone loss in postmenopausal women. However, the most recent evidence from a 10-year-follow-up observation confirmed that estrogen treatment for most newly menopausal women was safe and effective [42]. Therefore, the combination of HRT and exercise provides a feasible strategy for preventing fast bone loss during the first five years after menopause.

Recently, there was one meta-analysis by Zhang et al [43] that evaluated the additive effects of antiresorptive agents and exercise on BMD in adults. This study found that a combination of antiresorptive agents and exercise had additive effects on lumbar spine BMD. However, this meta-analysis was different from our study in many aspects. Firstly, the participants included in this review were adults, including both males and females, whereas our subjects were only postmenopausal women. It is known that man and estrogen-deficient women show different responses to the intervention of antiresorptive agents and exercise [17]. The mixture of males and females in study may ignore the different impacts of the combined intervention of antiresorptive agents and exercise on bone in different populations. Secondly, their participants included both healthy women and patients that had received medical treatment, such as heart and lung transplantation and immunosuppressive therapy, which potentially affected bone metabolism. Our subjects were mainly healthy postmenopausal women. The differing baseline characteristics of their participants probably increased the risk of bias. Additionally, compared with Zhang’s study, our meta-analysis includes a novel pharmacological strategy, isoflavone, which is recognized as a safe and effective treatment for preventing osteoporosis and appears to be less reported in the combination with exercise in preventing bone loss in postmenopausal women Our meta-analysis only shared a single randomized trial [23]with Zhang’s study and included eight extra clinical trials that were not included in Zhang’s meta-analysis.

In another meta-analysis, Martyn-St James et al [3] reported that the combination of high-intensity resistance training with HRT had additive effects on lumbar spine BMD in postmenopausal women. However, the results were limited by only including 3 RCTs.

Our findings indicated that impact exercise was sensitive to antiresorptive administration in preventing postmenopausal bone loss both at the spine and hips, whereas resistance training appeared to be less sensitive to the antiresorptive intervention. Borer [44] reports that the increment of BMD in elderly women following exercise are usually modest, which raises an important question what types of mechanical loading are optimum for improving bone health in postmenopausal women. It has been reported that adaptive skeletal response requires dynamic rather than static mechanical stimulation, and bone response is improved with brief but intermittent exercise [44, 45]. For the dynamic nature and simple style for performance, impact exercise plus an antiresorptive treatment may best benefit those postmenopausal women at high risk of fracture. Due to the between-study variation in pharmacological regimens, exercise interventions, sample sizes, and participant ages, we should view the non-significant effects of resistance training in conjunction with antiresorptive agents on preventing bone loss with caution.

The present findings are clinically significant because the additional increment of BMD induced by antiresorptive agents and exercise will effectively prevent bone loss and greatly benefit those postmenopausal women at risk of fracture. It has been reported that each 1 SD decrease in BMD is thought to be associated with a 10% increase in fracture risk [46]; findings from the present meta-analysis indicated that an additional increase in BMD could significantly affect the reduction in fracture risk at the spine and hips. The overall effects will be greater considering the benefits from exercise-related muscle mass increments, strength gained, joint flexibility and agility, and a well dynamic movement and balance [46].

Training-related injuries reported in the included studies were very low, indicating that the training exercises adopted by the participants were relatively safe for practice. However, relatively high withdrawal rates were reported in some trials, indicating that low rates of adherence to exercise interventions may generate a potential barrier to the improvement of postmenopausal bone health.

For the current meta-analysis, we conducted a systematic review to reduce the potential risk of bias. However, we did not present funnel plots to discuss publication bias, because the small number of study group comparisons available for the funnel plot interpretation was likely not sufficient to distinguish real asymmetry. Additionally, some authors have argued that some effect estimates, such as standardized mean difference, are naturally corrected with their standard errors, which probably generate spurious asymmetry in a funnel plot. Therefore, the subjective nature of visual interpretation of funnel plots seems to have limited use in the examination of publication bias in our meta-analysis.

The aspects of methodological quality, including randomization, blinding and statements on withdrawals, were assessed by a widely used instrument [35]. The quality score of the included trials was relatively low. According to the findings of Pildal [47], an inadequate concealment of allocation tends to overestimate the intervention effects; 2 of the included studies [14, 15] in our meta-analysis may have had inadequate allocation concealment and failed to avoid this type of bias. However, a more specific meta-analysis by Wood [48] found that the intervention effect size was overestimated when inadequate allocation concealment was present in trials with a subjective outcome but not in those with an objective outcome. Given that the primary outcomes in the included studies were objective measures, inadequate sequence generation might not pose much of a threat.

Three trials [28–30] included in our meta-analysis used double-blinding for pharmacological interventions; one trial [12] applied a single blinding for the measurer. No blinding methods were used for the exercise interventions in any of the studies. It has been reported that a lack of blinding is associated with exaggerated intervention effects [47]. However, this potential bias was lower for trials with objective outcomes compared with those with subjective outcomes. Given the objective nature of BMD measurements, the lack of blinding in most of the included studies may not have posed much of a threat towards bias. This is important because it is difficult to perform double blinding in exercise interventions.

An ITT analysis was used in 3 studies [28–30]; the other studies that were unable to provide a valid ITT strategy when attrition occurred were analyzed by a per-protocol approach. The ITT analysis is preferred because it is unbiased in addressing clinically relevant research questions; not all clinical trials qualified for an ITT analysis, which may induce a potential bias as a result of attrition failing to be accounted for.

Our meta-analysis provides definitive evidence that combining antiresorptive agents and excise generates additive effects on postmenopausal bone loss. The findings were clinically relevant and applicable in older women. However, there are limitations inherent in our meta-analysis. In all of the included trials, the BMD measurements were made with DXA. However, this may not be the optimal means to examine bone strength. Bone can adapt through both mineral materials and structure to increase mechanical loading [49]. It has been reported that BMD only accounts for approximately 60–70% of the variation in bone strength [50]; it does not account for other aspects of bone quality, such as microarchitecture. Therefore, BMD measurements may not well predict skeletal responses to antiresorptive treatment and exercise in postmenopausal women. Further studies conducted among postmenopausal women are needed to identify the material as well as structural changes that occur after antiresorptive administration and exercise interventions. From a clinical perspective, these adaptive processes are important because even small changes in bone geometry and structure can significantly improve bone strength in elderly women.

Our meta-analysis was also limited by the number of eligible clinical trials and the fact that the data were from highly selected samples of postmenopausal women of varying ages. Additionally, the relative low quality of some studies was also the limitation of our meta-analysis.

## Conclusions

In general, the present meta-analysis concluded that combining antiresorptive agents and exercise generated additive effects for preserving lumbar spine BMD in postmenopausal women. HRT combined with exercise training generated greater beneficial effects on lumbar spine and femoral neck BMD than the exercise-only intervention. Additionally, different exercise training modes show different sensitivities to antiresorptive treatments, with impact exercise having a greater sensitivity to antiresorptive agents in preventing postmenopausal bone loss. Our results provide synthesized evidence for the notion that antiresorptive agents can up-regulate the effects of mechanical loading on BMD in postmenopausal women. Therefore, to best increase the effects of exercise, combined protocols that integrate antiresorptive agents and exercise may be a feasible strategy for preventing postmenopausal bone loss. However, the limited number of eligible clinical trials and the poor quality of some studies remind us that further well-designed studies with large sample sizes are still needed.

## Supporting Information

S1 PRISMA Checklist PRISMA 2009 checklist in this meta-analysis. (DOC)
